# Prevalence of hypertension and its associated factors in Hawassa city administration, Southern Ethiopia: Community based cross-sectional study

**DOI:** 10.1371/journal.pone.0264679

**Published:** 2022-03-01

**Authors:** Tsegab Paulose, Zerish Zethu Nkosi, Misganu Endriyas

**Affiliations:** 1 Health Studies Department, University of South Africa Ethiopia, Regional Learning Center, Addis Ababa, Ethiopia; 2 Health Studies Department, University of South Africa, Pretoria, South Africa; 3 SNNPR Health Bureau, Hawassa, Ethiopia; ICMR-National Institute for Research in Tuberculosis: National Institute of Research in Tuberculosis, INDIA

## Abstract

**Background:**

In association with the epidemiological, nutritional and demographic transition, many research findings showed that the number of risk factors that leads to increased prevalence of hypertension in low and middle income countries like Ethiopia is increasing. Several urban specific studies conducted in Ethiopia showed varying prevalence of hypertension. The aim of this study was to determine prevalence of hypertension and to identify factors associated with hypertension in Hawassa city administration, Southern Ethiopia.

**Methods:**

A community-based cross sectional study was carried out in Hawassa city administration in 2017. A multi-stage sampling technique was used to select 612 study participants. Descriptive statistics was used to describe socio-demographic, behavioral and anthropometric variables. The economic status of household, ‘wealth index’, was constructed by running principal component analysis. Binary logistic regression analysis was performed to assess factors associated with hypertension at 95%CI.

**Results:**

The overall prevalence of hypertension was 21.2% (95% CI: 18.1–24.7), (24.5% for urban and 14.7% for peri-urban). About two fifths of hypertension cases (42.3%) were newly diagnosed with elevated blood pressure during data collection. Age, occupation, wealth status, consuming vegetables and animal fat, usual mode of transport, body mass index (BMI), family history of hypertension and existence of diabetes were associated with presence of hypertension at 95%CI. The average diastolic blood pressure for urban was 2.18mmHg higher than that of peri-urban groups (p-0.01).

**Conclusion:**

More than one fifth of study participants had hypertension and about two fifths of hypertension cases were newly diagnosed. Health communication should be strengthened focusing on identified risk factors and attention should be given to early detect and tackle the effects of hypertension in resource limited setting.

## Background

Elevated blood pressure (BP) or hypertension is a serious medical condition that significantly increases the risks of heart, brain, kidney and other diseases. According to World Health Organization (WHO) statistics, globally an estimated 1.13 billion people have hypertension, from which two-thirds are living in low- and middle-income countries [1].

Analysis of data from six countries showed that the prevalence of hypertension around the world has variations ranging from 3.4% to 78%, the lowest prevalence being among men in rural India and the highest prevalence in South Africa [2]. The prevalence also varies across country income groups and WHO regions. The WHO African Region has the highest prevalence of hypertension (27%) while the WHO Region of the Americas has the lowest prevalence of hypertension (18%). This increase in African region is due mainly to a rise in hypertension risk factors in those populations [1].

Different studies [3–12] reported different risk factors associated with hypertension. Some of these are age, alcohol, sex, obesity, smoking, physical inactivity, low fruit intake, chewing kchat, educational status, residence, meat consumption, diabetes, family history, marital status, occupation, income and salt intake.

In African countries like Cameroon and Tunisia, community-based multicenter and national cross-sectional studies conducted in major cities presented the overall prevalence of hypertension to be 47.5% and 35.1% respectively [13,14]. In sub-Saharan Africa, the prevention, detection, treatment and control of hypertension are suboptimal due to a combination of lack of resources and healthcare systems, absence of effective preventive strategies at population level, lack of sustainable drug therapy, and non-adherence to prescribed medications [15]. Rapid urbanization, adoption of unhealthy eating habits, and sedentary lifestyles are contributing for rapid increment of the problem [16,17] but majority of people are undiagnosed and only about a third of African adults with hypertension are aware of their condition [18].

A STEPS survey conducted in 2015 reported that the prevalence of hypertension was 15.6% (95% CI: 14.4–16.9%) [19]. A more recent systematic review and meta-analysis showed that overall pooled prevalence of hypertension in Ethiopia was 21.81% (95% CI: 19.20–24.42) [20]. Several urban specific studies conducted in Ethiopia showed varying level of hypertension. Some of studies and prevalence of hypertension reported were Dire Dawa 24.43% [3], Northwest Ethiopia (Dabat and Gonder) 27.9% [5], Jigjiga 28.3% [6], Addis Ababa 25% [7], Gondar 28.3% [8] and Durame 22.4% [9].

Besides the differences in prevalence of hypertension across towns in Ethiopia, there was lack of information on prevalence of hypertension in the study area comparing urban and peri-urban settings, including factors associated with hypertension. So, the aim of this study was to determine prevalence of hypertension and to identify factors associated hypertension in Hawassa city, Southern Ethiopia.

## Methods

**Fig 1:**
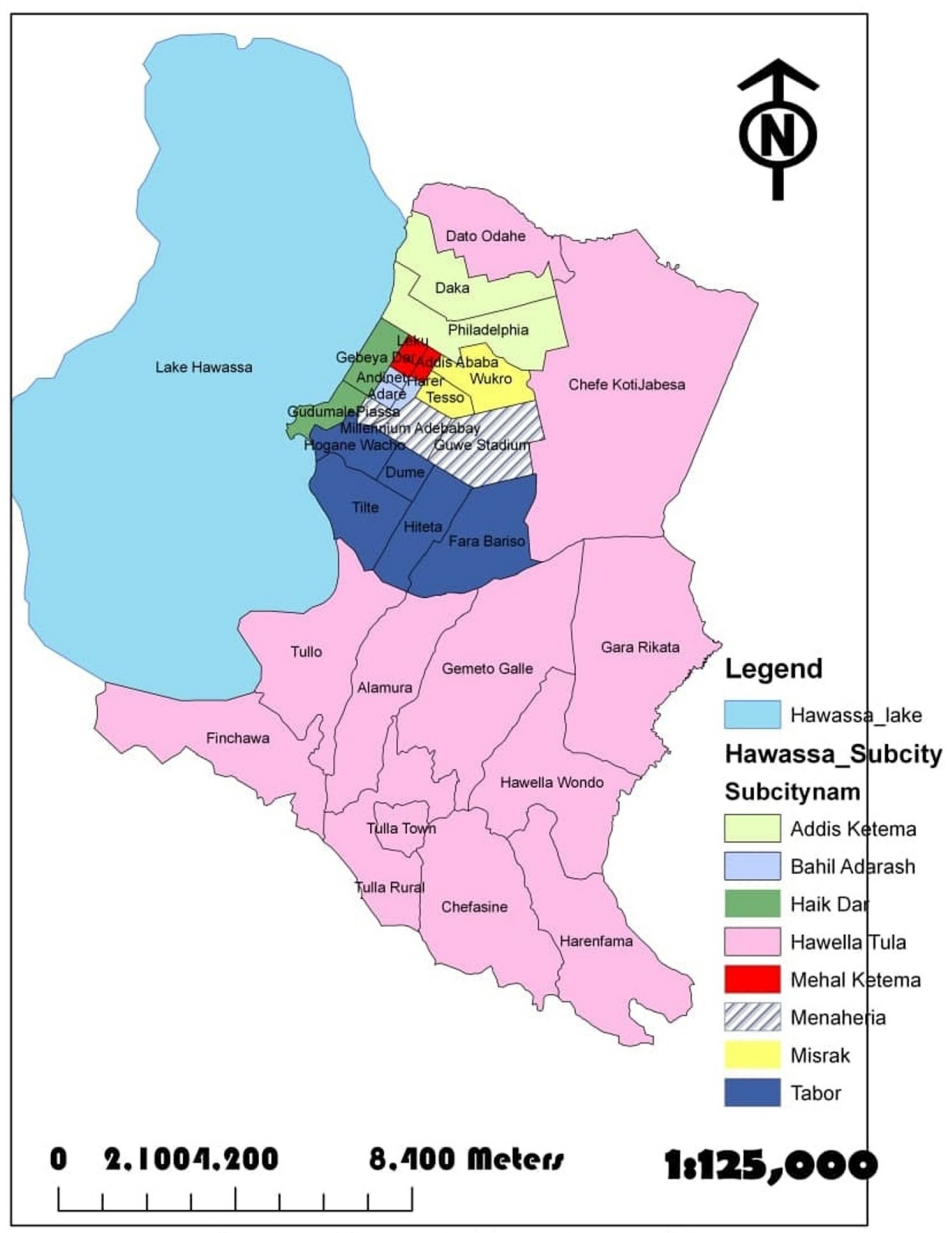
Administrative map of Hawassa city. Source: Development data collection and dissemination process, finance and economic development department, Hawasssa city administration.

A community-based cross sectional study was carried out in Hawassa city administration in 2017. Hawassa City (Fig 1) is located 273 km south of the capital, Addis Ababa via Debre Zeit and 1125 km north of Nairobi, Kenya. According to the population projection of Central Statistics Agency (CSA), Hawassa city administration had an estimated population of 351,567 in 2017 from which 170,510 were females and 181,057 were males. From the total population of the city, 250,777 were living in urban area while the remaining 100,790 were living in the peri-urban area. Urban area in the study setting refers to city while peri-urban area is partly urban and partially rural.

Sample size was calculated using single population formula considering 95% confidence interval, 5% margin of error, 80% power, proportion of 50% (to maximize sample size), 10% non-response rate and design effect of 1.5. Finally, the sample size was 633.

A multi-stage sampling technique was used to select the study participants. At first stage, three sub-cities (two urban and one peri-urban) were selected out of eight sub-cities in the city administration. At second stage, three kebeles from each sub-city, a total of nine kebeles were selected using simple random sampling. Kebeles are lowest administrative structures in the country. The estimated sample size was allocated proportionally to selected kebeles based on population size of kebeles. At third stage, households were selected from kebeles using simple random sampling. In the kebele, there are lists of households and this list was used as sampling frame. In households where there were more than one adult, random sampling method was used to select one adult. Residence of at least six months in the city and age of 30 or more years were considered as inclusion criteria.

Data was collected using a structured questionnaire which was organized based on review of similar literature and WHO STEP wise approach to surveillance of non-communicable diseases. The questionnaire comprised demographic and socio-economic variables, behavioral measurements, history of raised blood pressure and co-morbidity and physical measurements.

Content and criterion approaches were used to ensure validity in the study and reliability was ensured through pre-testing of the questionnaire in a setting other than the actual data collection was commenced. After pretest, leading questions were rephrased, order of questions were reshuffled, redundant questions were omitted, new questions were added, and questions which have similar concept and the same meaning were merged together.

Blood pressure was measured three times at the left upper arm of the seated subject using aneroid sphygmamometer of an appropriate size after the subject had rested for at least five minutes in a seated position and within 15 minutes interval. It was assured that the subjects have not consumed any hot beverages, such as tea or coffee, smoked cigarette or undertaken any vigorous-intensity physical activity within the last 30 minutes preceding the interview. In such cases, the measurement was postponed for 30 minutes. Finally, the last two measurements were considered to diagnose elevated blood pressure, considering average of the last two [21]. Hypertension was defined by considering WHO definition (≥140/90 mmHg and/or diastolic pressure ≥90 mmHg) [22] and/or being on medication of hypertension at the time of data collection. Anthropometric and blood pressures were measured by trained nurses. The height of the participant was measured at standing upright position on bare footed with 0.1 cm resolution and weight of participant wearing light cloth with 0.1 kilogram resolution.

Descriptive statistics like frequency, percentages, measures of central tendency (mean, median and mode) and standard deviations were performed to describe study participants. The economic status of household, ‘wealth index’, was constructed by running principal component analysis. Independent sample t-test was used to test mean difference of blood pressure between urban and peri-urban groups. Binary logistic regression was used to assess factors associated with hypertension. Variables that had p-value of 0.2 or less during bivariate analysis were considered for multivariable regression and finally, variables that had p-value of less than 0.05 in final model were reported as associated factors with adjusted odds ratio (AOR) and 95% CI.

Ethical clearance was obtained from the Research and Ethics Committee of University of South Africa (UNISA) (Reference number: REC-012714-039). Permission letter was obtained from Southern Nations Nationalities and Peoples’ Region health bureau and Hawassa city administration health department. Written consent was obtained from study participants. Respondents with elevated blood pressure were linked to healthcare.

## Results

### Socio-demographic characteristics

Out of the total 633 adults approached for interview and measurements, 612 agreed to fully participate in the study, yielding a response rate of 96.5%. About two thirds (66.7%) were from urban setting and about half (53.4%) were male. About two fifths (42.5%) were employed and majority, 500 (81.7%), were married. More than one third, 212 (34.6%), were overweight while 24 (3.9%) were obese (Table 1).

**Table 1:** Socio-demographic characteristics of respondents, Hawassa, 2017.

Variable	Categories	Frequency	Percent
Residence	Urban	408	66.7
	Peri-Urban	204	33.3
Sex	Male	327	53.4
	Female	285	46.6
Age	31–40	260	42.5
	41–50	152	24.8
	51–60	92	15.0
	61+	108	17.6
Marital status	Married	500	81.7
	Divorced/Widowed	83	13.6
	Single	29	4.7
Educational status	Cannot read and write	139	22.7
	Read and write only	98	16.0
	Primary education(1–8)	84	13.7
	Secondary education(9–12)	108	17.6
	Diploma and above	183	29.9
Occupation	Employee	199	32.5
	Daily-laborer	53	8.7
	Merchant	165	27.0
	House wife	118	19.3
	Retired	59	9.6
	Others	18	2.9
Wealth index	Lowest	170	27.8
	Second	103	16.8
	Middle	117	19.1
	Fourth	161	26.3
	Highest	61	10.0
BMI	Normal	366	59.8
	Under weight	10	1.6
	Over weight	212	34.6
	Obese	24	3.9

### Behavioral characteristics

Only about one out of ten (12.1%) and about one fourth (23.0%) reported that they have ever smoked cigarette and ever drunk alcohol respectively. Concerning diet balancing, nearly seven out of 10 (70.1%) reported that they consume fruits at least once per week while 524 (85.6%) consume vegetables at least once per week. In addition, about one third (30.7%) of respondents said that they eat animal meat at least once per week while 81 (13.2%) add additional salt on the top of prepared food. Regarding physical inactivity, near to half, 313 (49.9%), of respondents reported that their frequent mode of transport was motor driven vehicle while others use pedal bicycle and/or walk on foot (Table 2).

**Table 2:** Behavioral characteristics of study respondents, Hawassa, 2017.

Variable	Categories	Frequency	Percent
Ever smoke cigarette	No	538	87.9
	Yes	74	12.1
Ever drink alcohol	No	471	77.0
	Yes	141	23.0
Eat fruit at least once per week	No	183	29.9
	Yes	429	70.1
Eat vegetable at least once per week	No	88	14.4
	Yes	524	85.6
Eat animal meat at least once per week	No	424	69.3
	Yes	188	30.7
Use top added salt	No	531	86.8
	Yes	81	13.2
Usual mode of transport	On foot/pedal bicycle/	313	51.1
	Engine driven vehicle	299	48.9

### Non-modifiable risk factors

Eighty four (13.7%) respondents reported family history of hypertension. In addition, near to one out of ten (8.3%) reported that they were told presence of diabetes mellitus (DM).

### Prevalence of hypertension

The mean systolic blood pressure was 115.27 (SD = 11.09) mmHg, ranging from 80 to 150 while the mean of diastolic blood pressure was 75.75 (SD = 11.05) mmHg, ranging from 50 to 100. The overall prevalence of hypertension was 130 (21.2%) (95% CI: 18.1–24.7), (24.5% for urban and 14.7% for peri-urban), from which 55 out of 130 (42.3%) cases were newly diagnosed with elevated blood pressure while 75 were receiving medications of hypertension.

Concerning urban and peri-urban variations of blood pressures, independent t-test was used to test if there was differences in mean of blood pressures between groups. The test showed that there was no difference in mean of systolic blood pressure while there was difference in mean of diastolic blood pressure (t_481.436_ = 2.46, p = 0.01). The average diastolic blood pressure of urban was 2.18mmHg higher than that of peri-urban groups (95% CI: 0.44–3.93).

### Factors associated with hypertension

Binary logistic regression was used to assess factors associated with hypertension. After running bivariate and multivariable binary logistic regression, age, occupation, wealth status, consuming vegetables and animal meat, usual mode of transport, BMI, family history of hypertension and existence of DM were associated with presence of hypertension at 95%CI (Table 3).

**Table 3:** Bivariate and multivariable binary logistic regression of hypertension risk factors, Hawassa, 2017.

Variable	Category	Hypertension		Crude Odds Ratio 95% CI	AOR 95% CI*
		No No (%)	Yes No (%)		
Residence	Urban	308 (75.5)	100 (24.5)	1	
	Peri-urban	174 (85.3)	30 (14.7)	0.53 [0.34–0.83]	
Sex	Male	242 (74.5)	85 (26.5)	1	
	Female	240 (84.2)	45 (15.8)	0.53 [0.36–0.80]	
Age	31–40	234 (90.0)	26 (10.0)	1	
	41–50	116 (76.3)	36 (23.7)	1.79 [1.61–4.85]	2.42 [1.24–4.73]
	51–60	71 (77.2)	21 (22.8)	2.67 [1.41–5.01]	3.76 [1.70–8.31]
	≥ 61	61 (56.5)	47 (43.5)	6.93 [3.98–12.09]	7.85 [3.37–18.30]
Marital status	Married	392 (78.4)	108 (21.6)	1	
	Divorced/separated/widowed	65 (78.3)	18 (21.7)	1.00 [0.57–1.77]	
	Single	25 (86.2)	4 (13.8)	0.58 [0.19–1.71]	
Educational status	Cannot read and write	106 (76.3)	33 (23.7)	1	
	Read and write only	82 (83.7)	16 (16.3)	0.63 [0.32–1.22]	
	Primary (1–8)	68 (81.0)	16 (19.0)	0.76 [0.39–1.48]	
	Secondary (9–12)	86 (79.6)	22 (20.4)	0.82 [0.45–1.51]	
	Diploma and above	140 (76.5)	43 (23.5)	0.99 [0.59–1.66]	
Occupation	Employed	167 (83.9)	32 (16.1)	1	
	Daily-laborer	44 (83.0)	9 (17.0)	1.07 [0.47–2.40]	3.22 [1.01–10.29]
	Merchant	122 (73.9)	43 (26.1)	1.84 [1.10–3.07]	2.16 [1.01–4.63]
	House wife	96 (81.4)	22 (18.6)	1.19 [0.66–2.17]	2.72 [0.93–7.98]
	Retired	39 (66.1)	20 (22.9)	2.68 [1.38–5.17]	1.40 [0.46–4.27]
	Others	14 (77.8)	4 (22.2)	1.49 [0.46–4.82]	2.57 [0.54–12.14]
Wealth Index	Lowest	149 (87.6)	21 (12.4)	1	
	Second	79 (76.7)	24 (23.3)	2.26 [1.13–4.11]	2.98 [1.29–6.89]
	Middle	88 (75.2)	29 (24.8)	2.34 [1.26–4.35]	1.99 [0.82–4.83]
	Fourth	124 (77.0)	37 (23.0)	2.12 [1.18–3.80]	1.93 [0.76–4.91]
	Highest	42 (68.9)	19 (31.1)	3.21 [1.58–6.52]	2.45 [0.81–7.46]
Ever smoke cigarette	No	437 (81.2)	101 (18.8)	1	
	Yes	45 (60.8)	29 (39.2)	2.79 [1.67–4.66]	
Ever drink alcohol	No	392 (83.2)	79 (16.8)	1	
	Yes	90 (63.8)	51 (36.2)	2.81 [1.85–4.28]	
Eat fruit at least once per week	No	142 (77.6)	41 (22.4)	1	
	Yes	340 (79.3)	89 (20.7)	0.91 [0.60–1.38]	
Eat vegetable at least once per week	No	60 (68.2)	28 (31.8)	1	
	Yes	422 (80.5)	102 (19.5)	0.52 [0.32–0.85]	0.36 [0.18–0.73]
Eat animal fat at least once per week	No	355 (83.7)	69 (16.3)	1	
	Yes	127 (67.6)	61 (32.4)	2.47 [1.66–3.68]	1.83 [1.07–3.16]
Use top added salt	No	432 (81.4)	99 (18.6)	1	
	Yes	50 (61.7)	31 (38.3)	2.71 [1.64–4.45]	
Usual mode of transport	On foot/pedal bicycle/	262 (83.7)	51 (16.3)	1	
	Engine driven vehicle	220 (73.6)	79 (26.4)	1.85 [1.24–2.74]	1.71 [1.00–2.93]
BMI	Normal	317 (86.6)	49 (13.4)	1	
	Under weight	8 (80.0)	2 (20.0)	1.62 [0.33–7.84]	2.43 [0.41–14.33]
	Over weight	145 (68.4)	67 (31.6)	2.99 [1.97–4.54]	3.01 [1.77–5.10]
	Obese	12 (50.0)	12 (50.0)	6.47 [2.75–15.21]	12.25 [3.62–41.51]
Reported family history of hypertension	No	443 (83.9)	85 (16.1)	1	
	Yes	39 (46.4)	45 (53.6)	6.01 [3.69–9.79]	4.10 [2.10–8.01]
Reported presence of DM	No	463 (82.5)	98 (17.5)	1	
	Yes	19 (37.3)	32 (62.7)	7.96 [4.33–14.62]	4.37 [1.96–9.74]

*- cells with no significant associations were left blank to minimize texts in table.

Higher ages as compared to younger ages, daily laborers and merchants as compared to employed respondents, second class in wealth index as compared to lowest, obese as compared to normal BMI, consuming animal meat, not eating vegetables, usually using engine driven transport, family history of hypertension and presence of DM were factors associated with hypertension.

## Discussion

This study was done with the objective of determining prevalence of hypertension and its associated factors in Hawassa city, Southern Ethiopia. Key findings included that the prevalence of hypertension was 21.2% (24.5% for urban and 14.7% for peri-urban). Age, occupation, wealth status, consuming vegetables and animal fat, usual mode of transport, BMI, family history of hypertension and existence of DM were factors associated with presence of hypertension at 95% CI.

A systematic meta-analysis done in 2020 on prevalence of hypertension in Ethiopia reported that the overall pooled prevalence of hypertension to be 21.81% (95% CI: 19.20–24.42) [20]. Even though the prevalence of hypertension determined in this study was approximately comparable with that of meta-analysis report, it was slightly lower than most of studies done in urban settings of Ethiopia. Some of these studies and reported prevalence include Dire Dawa 24.43% [3], Northwest Ethiopia (Dabat and Gonder) 27.9% [5], Jigjiga 28.3% [6], Addis Ababa 25% [7], Gondar 28.3% [8] and Durame 22.4% [9]. Although hypertension was not significantly associated with permanent residence in this study at multivariable level analysis, the inclusion of peri-urban setting might have lowered the prevalence. That is, the lifestyle of peri-urban settings might have protective effect as evidences from Africa show that prevalence of hypertension is lower in some rural settings, including Ethiopia [23–26].

The study setting and the country in general have been implementing a policy prioritizing communicable diseases and little attention had been given to non-communicable diseases. In addition, the biased perception of the community that the non-communicable diseases are problems of rich is also contributing to the poor prevention measures and the prevalence of the disease is at significant level needing public intervention.

Concerning factors associated with hypertension, variables such as age, occupation, wealth status, consuming vegetables and animal fat, usual mode of transport, BMI, family history of hypertension and existence of DM were associated with having hypertension. These risk factors have been reported by different studies [3–12] in different areas.

Studies done on risk factors of hypertension reported that as age increases, the risk of hypertension increases [3–5,20,27,28]. Similarly, in this study, the likelihood of hypertension increased with advancing age. As age category increased from 31–40 to 41–50, 51–60 and above 60, the odds of having hypertension increased from 2.45 [AOR at 95% CI 1.24–4.73], 3.76 [AOR at 95% CI 1.70–8.31] and 7.85 [AOR at 95% CI 3.37–18.30] respectively. This could be due to the biological effect of increased arterial resistance because of arterial thickening at older age [29].

Concerning occupation, daily laborer and merchants were 3.22 and 2.16 times more likely to have hypertension as compared to employed respondents respectively. Study done in Dire Dawa, Ethiopia also reported office workers had lower odds of having hypertension than unemployed groups [3]. In this study, though employed groups had lower odds than daily laborers and merchants, there are some considerations to accept the association as we know that daily laborers do more physical activities than employed groups. First, the association was weak (p-0.049) and as a limitation, we don’t know types of activities performed by employed people included in the study.

Furthermore, individuals from second wealth class, having higher BMI, having family history of hypertension, having DM, commonly using motor driven vehicles, consuming animal meat and don’t consuming vegetables were more likely to have hypertension, which are also in line with above studies and other hypertension prevention recommendations [1,11,20,30].

Even though hypertension can be effectively controlled through appropriate lifestyle and cost-effective treatment, the disease response is minimal and high amount of people with the problem are not diagnosed [15,16,18]. In addition, awareness, treatment, and control of hypertension are very low in low-income and middle-income countries [31].

In this study, despite high prevalence of the disease in the community, as much as about two fifths of total hypertension cases were newly diagnosed during data collection and it is important to expect the number of cases that can be identified if mass screening is conducted. In the study setting, the response to non-communicable diseases including hypertension is based on passive surveillance and individual oriented. That is, people come to health facilities when they become sick at late stage and cares are given to those who are coming to the facilities. Based on the cases yield from this study, we recommend outreach services like mass screening and focusing on mass population. We also recommend health system in resource limited setting to focus on promoting and prioritizing lifestyle change, early diagnosis and control of the disease.

Even though this study assessed prevalence and risk factors of hypertension in the city considering urban and peri-urban settings in its sampling, limitations of this study were it included permanent residents of the city administration who lived in the area for more than six months and age greater than 30 years old. In addition, it did not measure amount of alcohol, cigarette, fruits, vegetables, fats and salts consumed in measurable units. Finally, the study did not capture details on medical care of those who were taking hypertension medications.

## Conclusion

More than one fifth of study participants had hypertension and about two fifths of hypertension cases were newly diagnosed. Age, occupation, wealth status, consuming vegetables and animal fat, usual mode of transport, BMI, family history of hypertension and existence of DM were associated with presence of hypertension.

Health communication should be strengthened focusing on identified risk factors and attention should be given to early detect the medical condition and tackle effects of hypertension in resource limited setting.

## Supporting information

S1 File (SAV)

S1 Questionnaire (DOCX)

## Abbreviations

BMI: Body mass index
BP: Blood pressure
DM: Diabetes mellitus
HTN: Hypertension
