# Supplementary material for: Prevalence of hypertension and its associated factors in Hawassa city administration, Southern Ethiopia: Community based cross-sectional study
Source: PLoS One. 2022 Mar 1;17(3):e0264679. doi: 10.1371/journal.pone.0264679 (PMC8887727; doi:10.1371/journal.pone.0264679)
Supplement: S1 Questionnaire — (DOCX) [file pone.0264679.s002.docx]

**Annexure O: Questionnaire English version**

University of South Africa College of human sciences health studies department

Greeting

My name is ___________________ currently I am collecting data for the research that will be conducted by University of South Africa PHD student Tsegab Paulose in accordance with the requirements for the degree of Doctor of literature and philosophy, among adults (>30 years age) in Hawassa city administration on Hypertension.

As you are permanent dweller of Hawassa city you are randomly selected to be part of the study; for your involvement have significant value to the study.

The study includes asking you few questions on hypertension and taking measurements of your blood pressure, weight and height. It takes us about 60 minutes. The information collected from you will be kept confidential and will not be accessed by anyone except the principal investigator and also your name will not be included in the information.

Your participation in this research study is voluntary. You may choose to participate or not and you may withdraw your consent to participate at any time without losing any of your right. I kindly request you to participate in this study?

I have been briefly informed about the study and I clearly understood the objective and agreed to take part in the study as an Interviewee_______

But do not agreed to take part in the study as an Interviewee_______

Name of kebele ______________

Signature of data collector ___________________

**Section I) Demographic and Socio -Economic data**

| No | Questions | Response | Remark |
| --- | --- | --- | --- |
| 101 | Permanent residence | 1. Urban  2. Peri- Urban |  |
| 102 | Sex | 1. Male  2. Female |  |
| 103 | How old are you? | Age in completed years ______ |  |
| 104 | What is your current marital status?  Circle the response. | 1. Single  2. Married  3. Divorced  4. Widowed |  |
| 105 | What is your highest educational status? /In the completed year/ | 1 Cannot read and write  2 Read and write only  3 primary education(1-8)  4.secondary education(9-12)  5. diploma and above |  |
| 106 | What is your religious affiliation? | 1. Protestant  2. Orthodox  3. Catholic  4. Muslim  5. Other |  |
| 107 | Ethnicity | 1. Sidama  2. Walayita  3. Kembata  4. Gurage  5. Amahra  6.Oromo  7. Hadiya  8. Other |  |
| 108 | What is your current occupation? | 1. Employee(GO/NGO)  2. Daily-laborer  3. Merchant  4. House wife  5. Retired  6. others(specify) |  |
| 109 | What is your family size? | ________ |  |
| 110 | How much is your family average total monthly income? | Approximate_________Eth.birr |  |
| 111 | Owner ship of house? | 1 Rental  2 Personal |  |
| 112 | Does your household have: |  |  |
|  | Electricity | 1.Yes 2.No |  |
|  | Radio | 1.Yes 2.No |  |
|  | Television | 1.Yes 2.No |  |
|  | Refrigerator | 1.Yes 2.No |  |
|  | Mobile phone | 1.Yes 2.No |  |
|  | Bicycle | 1.Yes 2.No |  |
|  | Motor bike | 1.Yes 2.No |  |
|  | Car | 1.Yes 2.No |  |
|  | Table | 1.Yes 2.No |  |
|  | Chair | 1.Yes 2.No |  |
|  | Bed with cotton/sponge/spring mattress | 1.Yes 2.No |  |
|  | An electric mitad | 1.Yes 2.No |  |
|  | A kerosene lamp/pressure lamp | 1.Yes 2.No |  |
|  | Bank or microfinance saving account | 1.Yes 2.No |  |

**Section II) Behavioural Measurements**

| Cigarettes use | | | |
| --- | --- | --- | --- |
| No. | Question | Response | Remark |
| 401 | Have you ever smoked cigarettes? | 1 Yes  2 no |  |
| 402 | Do you smoke currently? | 1 Yes  2 no |  |
| 403 | If yes for Q402 how frequent, do you smoke cigarettes? | 1. daily  2. 5-6 days per week  3. 3-4 days per week  4. 1-2 days per week |  |
| 404 | On average, how much cigarettes do you smoke in this days? | _____ Number |  |
| 405 | For how long have you been smoking? | _____ In Years |  |
| Alcohol consumption | | | |
| 406 | Have you ever consumed an alcoholic drink | 1yes  2 no |  |
| 407 | Do you drink alcoholic drink currently? | 1 Yes  2 no |  |
| 408 | If yes for Q407 how frequent do you drink alcoholic drink? | 1. daily  2. 5-6 days per week  3. 3-4 days per week  4. 1-2 days per week |  |
| 409 | How much glass at one time?  (300ml for beer, tea cup for local drink) | _____Number |  |
| 410 | For how long have you been drinking? | _____ Years |  |
| Chat chewing | | | |
| 411 | Have you ever chewed chat | 1 Yes  2 no |  |
| 412 | Do you chew chat currently? | 1 Yes  2 no |  |
| 413 | If yes for Q412 how frequent do you chew chat? | 1. daily  2. 5-6 days per week  3. 3-4 days per week  4. 1-2 days per week |  |
| 414 | For how long have you been chewing chat? | _____In Years |  |
| Coffee consumption | | | |
| 415 | Do you drink coffee | 1. Yes  2. No |  |
| 416 | If yes for Q415 how frequent do you drink coffee | 1. daily  2. 5-6 days per week  3. 3-4 days per week  4. 1-2 days per week |  |
| 417 | How many cups | _____cup in number |  |
| 418 | What do you use to give taste when you are drinking coffee | 1. Salt  2. sugar  3. No thing |  |
| Diet | | | |
| 419 | In which time of serving do you eat more? | 1. Breakfast  2. Launch  3. Dinner  4. Snacks |  |
| 420 | Do you eat fruit? | 1. Yes  2. No |  |
| 421 | If yes for Q420 how many days do you eat fruit in a week? | 1. daily  2. 5-6 days per week  3. 3-4 days per week  4. 1-2 days per week |  |
| 422 | Do you eat vegetables? | 1. Yes  2. No |  |
| 423 | If yes for Q422 how many days do you eat vegetables in a week? | 1. daily  2. 5-6 days per week  3. 3-4 days per week  4. 1-2 days per week |  |
| 424 | Do you eat animal fat (butter, fatty meat)? | 1. Yes  2. No |  |
| 425 | How many days do you eat animal fat in a week? | 1. daily  2. 5-6 days per week  3. 3-4 days per week  4. 1-2 days per week |  |
| Salt consumption | | | |
| 426 | Do you use additional top added salt on plate after food is prepared with sufficient amount of salt? | 1 yes  2 no |  |
| Physical activity | | | |
| 427 | Does your work involve vigorous-intensity activity that causes large increases in breathing or heart rate like (carrying or lifting heavy loads, digging or construction work) for at least 10 minutes continuously? | 1 yes  2 no |  |
| 428 | In a typical week, on how many days do you do vigorous-intensity activities as part of your work? | 1. daily  2. 5-6 days per week  3. 3-4 days per week  4. 1-2 days per week |  |
| Travel to and from places | | |  |
| 429 | Do you walk for at least 10 minutes a day continuously to get to and from places? | 1 yes  2 no |  |
| 430 | In a typical week, many days do you walk for at least 10 minutes continuously to get to and from places? | 1. daily  2. 5-6 days per week  3. 3-4 days per week  4. 1-2 days per week |  |
| 431 | What do you use to go and come from place to place | 1.On foot  2. bicycle  3. engine using vehicle |  |
| Recreational activities | | | |
| 432 | Do you do any vigorous-intensity sports, fitness or recreational (leisure) activities that cause large increases in breathing or heart rate like [running or football] for at least 10 minutes continuously? | 1 yes  2 no |  |
| 433 | In a typical week, on how many days do you do vigorous-intensity sports, fitness or recreational (leisure) activities? | 1. daily  2. 5-6 days per week  3. 3-4 days per week  4. 1-2 days per week |  |
| **Section V) History of raised Blood Pressure and co-morbidity** | | | |
| No. | Question | Response | Remark |
| 501 | Have you ever had your blood pressure measured by a doctor or other health worker? | 1 yes  2 no |  |
| 502 | Have you ever been told by a doctor or other health worker that you have hypertension? | 1 yes  2 no |  |
| 503 | Are you currently receiving any medication, treatments/advice for hypertension prescribed by a doctor or other health worker | 1 yes  2 no |  |
| 504 | Is there anyone from your family (father, mother or siblings) who have history of hypertension | 1 yes  2 no |  |
| 505 | Have you ever been told by a doctor or health worker that you have diabetes? | 1 yes  2 no |  |

**Section VI) Physical measurements**

| Height and Weight | | | |
| --- | --- | --- | --- |
| No. | Question | Response | Remark |
| 601 | Height in cm | ________cm |  |
| 602 | Weight in kg | _______kg |  |
| Blood Pressure with 15 minute interval | | | |
| 603 | Reading 1 | ____Systolic ( mmHg) |  |
|  |  | ____Diastolic (mmHg) |  |
| 604 | Reading 2 | ___Systolic ( mmHg) |  |
|  |  | ___Diastolic (mmHg) |  |
| 605 | Reading 3 | ___Systolic ( mmHg) |  |
|  |  | ___Diastolic (mmHg) |  |

Thank you!

**Annexure P: Questionnaire Amharic version**

University of South Africa College of human sciences health studies department

ሰላም

እኔ ……………………………………….………በደቡብ አፍሪካ ዩኒቨርስቲ በሰብዓዊና ጤና ሳይንስ የትምህርት ክፍል የዶክትሬት ዲግሪ ተማሪ ለሆኑት ፀጋአብ ጳውሎስ እድሜያቸው ከ30 አመት በላይ በሆኑ የሀዋሳ ከተማ ነዋሪዎች ላይ በደም ግፊት ዙሪያ ለሚያደርጉት ምርምርና ጥናት እንዲረዳ መረጃ ለማሰባሰብ የተመደብኩ ጤና ባለሙያ ነኝ::

እርሶም የከተማው ነዋሪ በመሆንዎ በዚህ ጥናት ላይ መሳተፍዎ ለጥናቱ ውጤታማነት ከፍተኛ አስተዋጽኦ ያደርጋል፡፡ በጥናቱም መሰረት የደም ግፊትን በተመለከተ ዉስን ጥያቄዎች ከመካተታቸውም በተጨማሪ የደም ግፊትዎ ፤ክብደትዎና ቁመትዎ ይለካል፡፡አጠቃላይ እስከ 60 ደቂቃዎችን የምንጠቀም ሲሆን ማንኛውም የሚሰጡት የግሎ መረጃ ሚስጥራዊነቱ የተጠበቀ እና ከአጥኙዉ ውጭ ለማንም የማይሰጥ እና ስሞትም የማይጠቀስ ይሆናል፡፡የእርሶም ተሳታፊነት በበጎ ፈቃድ ላይ ብቻ የተመሰረተ ሲሆን ባልፈለጉም ጊዜ ማቋረጥ ይችላሉ፤ ስለዚህ እንዲሳተፉ በትህትና እንጠይቃለሁ?

ከላይ የተገለፀዉን ማብራሪያ ተረድቻለዉ

በጥናቱ ለመሳተፍ መስማማቴን በፊርማ አረጋግጣለሁ ……………………………………

ነገር ግን በጥናቱ ለመሳተፍ አልስማማም …………………………………

የቀበሌው ስም………

የመጠይቁ መለያ ቁጥር________________

| **ክፍል 1፡ ኢኮኖሚያዊና ማህበራዊ መረጃዎ** | | | |
| --- | --- | --- | --- |
| ቁጥር | ጥያቄዎች | ምላሽ | ምርመራ |
| 101 | ቋሚ የመኖሪያ ቦታ | 1. ከተማ  2. ከፊል ከተማ |  |
| 102 | ጾታ | 1. ወንድ  2. ሴት |  |
| 103 | እድሜ | _______ዓመት |  |
| 104 | የጋብቻ ሁኔታ | 1. ያላገባ/ች  2. ያገባ/ች  3. ፍች የፈጸመ/ች  4. ባል/ሚስት በህይወት የሌለ |  |
| 105 | የትምህርት ደረጃ  /ባለፈዉ አመት ያጠናቀቁት የትምህርት ደረጃ/ | 1.መጻፍና ማንበብ የማይችል/የማትችል/  2.መጻፍና ማንበብ ብቻ የሚችል/የምትችል/  3.አንደኛ ደረጃ (1-8)  4.ሁለተኛ ደረጃ (9-12)  5.ዲፕሎማ እና ከዚያ በላይ |  |
| 106 | ሃይማኖት | 1.ፕሮቴስታንት  2.ኦርቶዶክስ  3.ካቶሊክ  4.ሙስሊም  5.ሌላ(ይግለጹ)....................... |  |
| 107 | ብሄር | 1. ሲዳማ  2. ወላይታ  3. ከምባታ  4. ጉራጌ  5. አማራ  6. ኦሮሞ  7. ሀድያ  8. ሌላ (ይግለጹ)____________ |  |
| 108 | የስራ ሁኔታ | 1.ተቀጥሮ የሚሰራ (በመንግስት/በግል)  2.የቀን ሰራተኛ  3. ነጋዴ  4. የቤት እመቤት  5. ጡረተኛ  6. ሌላ(ይግለጹ) |  |
| 109 | የቤተሰብ ብዛት በቁጥር | ________ |  |
| 110 | ወርሃዊ የቤተሰብ ገቢ በአማካይ በብር | በአማካይ_________ብር |  |
| 111 | የሚኖሩበት ቤት ባለቤትነት | 1የኪራይ  2 የግል |  |
| 112 | ቀጥሎ ከሚጠቀሱት ውስጥ በቤትዎ የሚገኙት አለ የማይገኙትን የለም በማለት ይመልሱ |  |  |
|  | ኤልክትሪክ/መብራት መስመር | 1.አለ 2.የለም |  |
|  | ሬዲዮ | 1.አለ 2.የለም |  |
|  | ቴሌቪዥን | 1.አለ 2.የለም |  |
|  | ፍሪጅ | 1.አለ 2.የለም |  |
|  | ስልክ /ሞባይል ወይም መደበኛ/ | 1.አለ 2.የለም |  |
|  | ብስክሌት | 1.አለ 2.የለም |  |
|  | ሞተር ሳይክል | 1.አለ 2.የለም |  |
|  | መኪና | 1.አለ 2.የለም |  |
|  | ጠረጴዛ | 1.አለ 2.የለም |  |
|  | ወንበር | 1.አለ 2.የለም |  |
|  | አልጋ ከፍራሽ ጋር | 1.አለ 2.የለም |  |
|  | የኤልክትሪክ ምጣድ | 1.አለ 2.የለም |  |
|  | ቡታ ጋዝ /የኤሌክትሪክ ምግብ ማብሰያ | 1.አለ 2.የለም |  |
|  | ባንክ /ማይክሮ ሲስተምስ አካውንት | 1.አለ 2.የለም |  |

**ክፍል 2: የባህሪይ መመዘኛዎች**

| ሲጋራ አጠቃቀም | | | |
| --- | --- | --- | --- |
| ቁጥር | ጥያቄ | ምላሽ | ምርመራ |
| 401 | ሲጋራ አጭሰው ያውቃሉ? | 1አዎ  2 አላውቅም |  |
| 402 | በአሁኑ ጊዜ ሲጋራ ያጨሳሉ? | 1አዎ  2 አላጨስም |  |
| 403 | ለጥያቄ 402 ምላሽዎ አዎ ከሆነ በምን ያህል ጊዜ ውስጥያጨሳሉ? | 1. በየቀኑ  2. በሳምንት ከ5-6 ቀናት  3. በሳምንት ከ3-4 ቀናት  4. በሳምንት ከ1-2ቀናት |  |
| 404 | በአማካይ በቀን ምን ያህል ሲጋር | _____በቁጥር |  |
| 405 | ያጨሳሉ ለምን ያህል ጊዜ አጭሰዋል? | _____ በአመት |  |
| አልኮል አጠቃቀም | | | |
| 406 | አልኮል ነክ መጠጦችን ጠጥተው ያውቃሉ? | 1አዎ  2 አላውቅም |  |
| 407 | በአሁኑ ጊዜ አልኮል ይጠጣሉ? | 1አዎ  2 አልጠጣም |  |
| 408 | ለጥያቄ 407 ምላሽዎ አዎ ከሆነ በምን ያህል ጊዜ ውስጥ ነዉ አልኮል መጠጥ የሚወስዱት? | 1. በየቀኑ  2. በሳምንት ከ5-6 ቀናት  3. በሳምንት ከ3-4 ቀናት  4. በሳምንት ከ1-2ቀናት |  |
| 409 | በአንድ ጊዜ ምን ያህል ጠርሙስ/ብርጭቆ ይጠጣሉ (300ሚሊ ለቢራ እና የሻይ ሲኒ ለባህላዊ መጠጥ) | _____ በቁጥር |  |
| 410 | ለምን ያህል ጊዜ ነዉ የጠጡት? | _____በአመት |  |
| ጫት መቃም | | | |
| 411 | ጫት ቅመው ያውቃሉ | 1አዎ  2 አላውቅም |  |
| 412 | በአሁኑ ጊዜ ጫት ይቅማሉ | 1አዎ  2 አልቅምም |  |
| 413 | ለጥያቄ 412 ምላሽዎ አዎ ከሆነ በምን ያህል ጊዜ ውስጥ ነዉ ጫት የሚቅሙት | 1. በየቀኑ  2. በሳምንት ከ5-6 ቀናት  3. በሳምንት ከ3-4 ቀናት  4. በሳምንት ከ1-2ቀናት |  |
| 414 | ለምን ያህል ጊዜ ጫት ቅመዋል? | _____በአመታት |  |
| ቡና አጠቃቀም | | | |
| 415 | ቡና ይጠጣሉ | 1. እጠጣለዉ  2. አልጠጣም |  |
| 416 | ለጥያቄ 415 ምላሽዎ አዎ ከሆነ በምን ያህል ጊዜ ውስጥ ነዉ የሚጠጡት | 1. በየቀኑ  2. በሳምንት ከ5-6 ቀናት  3. በሳምንት ከ3-4 ቀናት  4. በሳምንት ከ1-2ቀናት |  |
| 417 | ምን ያህል ሲኒ | _____ሲኒ በቁጥር |  |
| 418 | ቡና የሚጠጡት ምን ጨምሮ ነው? | 1. ጨው  2. ስካር  3. ባዶ |  |
| አመጋገብ | | | |
| 419 | በየትኛው የምግብ ሰአት ነው በአንጻሩ በብዛት የሚመገብት? | 1. በቁርስ ሰዓት  2. በምሳ ሰዓት  3. በእራት ሰዓት  4. በመክሰስ ሰዓት |  |
| 420 | ፍራፍሬዎችን ይመገባሉ? | 1 አዎ  2 አልመገብም |  |
| 421 | ለጥያቄ 420 ምላሽዎ አዎ ከሆነ በምን ያህል ጊዜ ውስጥ ነዉ ፍራፍሬዎችን የሚመገብት | 1. በየቀኑ  2. በሳምንት ከ5-6 ቀናት  3. በሳምንት ከ3-4 ቀናት  4. በሳምንት ከ1-2ቀናት |  |
| 422 | አትክልት ይመገባሉ | 1 አዎ  2 አልመገብም |  |
| 423 | ለጥያቄ 422 ምላሽዎ አዎ ከሆነ በምን ያህል ጊዜ ውስጥ ነዉ አትክልት የሚመገብት | 1. በየቀኑ  2. በሳምንት ከ5-6 ቀናት  3. በሳምንት ከ3-4 ቀናት  4. በሳምንት ከ1-2ቀናት |  |
| 424 | ቅቤና ጮማ የበዛበት ምግቦችን ይመገባሉ | 1 አዎ  2 አልመገብም |  |
| 425 | ለጥያቄ 424 ምላሽዎ አዎ ከሆነ በምን ያህል ጊዜ ውስጥ ነዉ አትክልት የሚመገብት | 1. በየቀኑ  2. በሳምንት ከ5-6 ቀናት  3. በሳምንት ከ3-4 ቀናት  4. በሳምንት ከ1-2ቀናት |  |
| የጨው አጠቃቀም | | | |
| 426 | በምግብ ውስጥ ከተጨመረው በላይ ሌላ ጨው ጨምረው ያበላሉ | 1 አዎ  2 አልመገብም |  |
| የሰውነት እንቅስቃሴ ሁኔታ | | | |
| 427 | በየዕለቱ የሚሰሩት ስራ ቢያንስ ለ10 ደቂቃ እንቅስቃሴ እና ጉልበት የሚጠይቅ ነው? | 1 አዎ  2 አይደለም |  |
| 428 | በሳምንት ለምን ያህል ጊዜ የጉልበት ስራ ይሰራሉ? | 1. በየቀኑ  2. በሳምንት ከ5-6 ቀናት  3. በሳምንት ከ3-4 ቀናት  4. በሳምንት ከ1-2ቀናት |  |
| ከቦታ ቦታ መንቀሳቀስ | | |  |
| 429 | በቀን ቢያንስ ለ10 ደቂቃ ከቦታ ቦታ ይንቀሳቀሳሉ | 1 አዎ  2 አልንቀሳቀስም |  |
| 430 | በአንድ ሳምንት ውስጥ ቢያንስ ለ10 ደቂቃ ያህል ምን ያህል ቀናት ይራመዳሉ? | 1. በየቀኑ  2. በሳምንት ከ5-6 ቀናት  3. በሳምንት ከ3-4 ቀናት  4. በሳምንት ከ1-2ቀናት |  |
| 431 | የሚጠቀሙት ትራንስፖርት አይነት | 1.በእግር  2.ቢስክሌት  3.ተሽከርካሪ |  |
| የመዝናኛ እንቅስቃሴ | | | |
| 432 | ጉልበት የሚጠይቅ የአካል ብቃት እንቅስቃሴ(ስፖርት)በማድረግ ነው ሚዝናኑት? | 1 አዎ  2 አልሰራም |  |
| 433 | በሳምንት ውስጥ ምን ያህል ጊዜ የአካል ብቃት እንቅስቃሴ ያደርጋሉ ወይስይዝናናሉ(በስፖርት) | 1. በየቀኑ  2. በሳምንት ከ5-6 ቀናት  3. በሳምንት ከ3-4 ቀናት  4. በሳምንት ከ1-2ቀናት |  |
| **ክፍል አምስት) ከደም ግፊት ጋር የተያያዘ ታሪክ እና ተዛማጁ በሽታዎች** | | | |
| ቁጥር | ጥያቄ | ምላሽ | ምርመራ |
| 501 | የደም ግፊትዎን በሀኪም ወይም በሌላ የጤና ባለሙያ ተለክተው ያውቃሉ? | 1 አዎ  2 አላውቅም |  |
| 502 | የደም ግፊት መጨመር አለቦት ተብለው ተነግሮዎታል? | 1 አዎ  2 አላውቅም |  |
| 503 | በአሁኑ ጊዜ በሀኪም ወይም በሌላ የጤና ባለሙያ የታዘዘልዎት ለደም ግፊት መጨመር መቆጣጠሪያ የሚወስዱት መድሀኒት ወይም ምክርና ህክምና አለ? | 1 አዎ  2 አልጠቀምም |  |
| 504 | በቤተሰብዎ መካከል የደም ግፊት መጨመር ያለበት ሰው አለ? | 1 አዎ  2 አላውቅም |  |
| 505 | የስኳር በሽታ አለብዎት ተብሎ በሀኪም ወይም በሌላ የጤና ባለሙያ ተነግሮት ያውቃል | 1 አዎ  2 አላውቅም |  |

**ክፍል ስድስት የሰውነት ልኬት**

| ቁመት እና ክብደት | | | |
| --- | --- | --- | --- |
| ቁጥር | ጥያቄ | ምላሽ | ምርመራ |
| 601 | ቁመት | ________ሳሜ |  |
| 602 | ክብደት | _______ኪ.ግ |  |
| የደም ግፊት ልኬት በ15 ደቂቃ ልዩነት | | | |
| 603 | ንባብ 1 | ____ሲስቶሊክ |  |
|  |  | ____ዳያስቶሊክ |  |
| 604 | ንባብ 2 | ____ሲስቶሊክ |  |
|  |  | ____ዳያስቶሊክ |  |
| 605 | ንባብ 3 | ____ሲስቶሊክ |  |
|  |  | ____ዳያስቶሊክ |  |

አመሰግናለዉ!
